# Motion-preserving treatment of unstable atlas fracture: transoral anterior C1-ring osteosynthesis using a laminoplasty plate

**DOI:** 10.1186/s12891-020-03575-w

**Published:** 2020-08-12

**Authors:** Xiaobao Zou, Beiping Ouyang, Binbin Wang, Haozhi Yang, Su Ge, Yuyue Chen, Ling Ni, Shuang Zhang, Hong Xia, Zenghui Wu, Xiangyang Ma

**Affiliations:** 1 The First School of Clinical Medicine, Southern Medical University, No.1838 North of Guangzhou Road, Guangzhou, 510515 People’s Republic of China; 2 Department of Orthopedics, General Hospital of Southern Theatre Command of PLA, 111 Liuhua Road, Guangzhou, 510010 People’s Republic of China

**Keywords:** Transoral approach, Atlas fracture, Unstable fracture, C1-ring osteosynthesis, Open reduction, Internal fixation

## Abstract

**Background:**

C1-ring osteosynthesis is a valid alternative to posterior C1–C2 or C0–C2 fusion to preserve important C1–C2 motion in the treatment of unstable atlas fractures. Nevertheless, the fixation instruments used in current studies for transoral anterior C1-ring osteosynthesis were not suitable for anterior anatomy of the atlas or did not have reduction mechanism. We therefore present this report to investigate preliminary clinical effects of transoral anterior C1-ring osteosynthesis using a laminoplasty plate in unstable atlas fractures.

**Methods:**

From January 2014 to December 2017, 13 patients with unstable atlas fractures were retrospectively reviewed. All patients were treated with transoral anterior C1-ring osteosynthesis using a laminoplasty plate. Pre- and postoperative images were obtained to assess reduction of the fracture, internal fixation placement, and bone union. Neurological function, range of motion, and pain levels were evaluated clinically on follow-up.

**Results:**

The surgeries were successfully performed in all cases. The average follow-up duration was 16.6 ± 4.4 months (range 12–24 months). One patient suffered screw loosening after operation and underwent replacement operation subsequently. Satisfactory clinical outcomes were achieved in all patients with ideal fracture reduction, reliable plate placement, well-preserved range of motion, and neck pain alleviation. All patients achieved bone union of fractures without loss of reduction or implant failure or C1–C2 instability during the follow-up. No vascular or neurological complication was noted during the operation and follow-up.

**Conclusions:**

Transoral anterior C1-ring osteosynthesis using a laminoplasty plate is a effective surgical treatment for unstable atlas fractures. This technique has a ingenious reduction mechanism, and can provide satisfactory bone union and preservation of C1–C2 motion.

## Background

Atlas fractures are not rare in cervical spine injury, account for 25% of craniocervical injuries, 2–13% of cervical injuries, 1–2% of all spinal injuries, and less likely to cause neurological deficits [1, 2]. The stability of atlas fractures is determined by the structural integrity of the transverse atlantal ligament (TAL) and the extent of fracture [3, 4]. The treatment of unstable atlas fractures is still controversial. Nonsurgical treatments of unstable atlas fractures have the disadvantages of deficient reduction and high rates of nonunion, and even neurological damage [5]. Although surgical treatments with C1–C2 or C0–C2 fusion can achieve satisfactory stability and bone fusion in unstable atlas fractures, the normal C1–C2 motion is lost [6]. Presently, C1-ring osteosynthesis is a well-known and effective management for unstable atlas fractures to preserve important C1–C2 motion [2]. But, the devices used in previous researches for transoral anterior C1-ring osteosynthesis were not suitable for anterior anatomy of the atlas or did not have reduction mechanism [7–9]. In this study, we retrospectively analyzed the clinical data of 13 patients with unstable atlas fracture who were treated by transoral anterior C1-ring osteosynthesis using a laminoplasty plate presenting with a ingenious reduction procedure, and evaluated the preliminary effects of this technique.

## Methods

### Patients

From January 2014 to December 2017, 13 consecutive patients with unstable atlas fractures were recruited (Table 1). Transoral anterior C1-ring osteosynthesis using a laminoplasty plate was performed in each case. Patients included 6 men and 7 women with a mean age of 47.8 years (ranging from 32 to 66 years). The causes of injury were falling (5 cases) and motor vehicle accident (8 cases). All patients were conscious and cooperative and presented with neck pain and stiffness without neurological symptoms. Routine preoperative open-mouth, anteroposterior and lateral radiographs, computed tomography (CT) with 3-dimensional reconstructions, and magnetic resonance imaging (MRI) were performed in each case. All cases in this study were unstable with combined fractures of the anterior and posterior atlantal arches based on multidetector CT, which were type II fractures according to Landells and Van Peteghem’s classification system [10]. No patients had anterior or ratatory atlantoaxial dislocation. Nine patients had injuries of the TAL shown on MRI or CT images. Dickman type I and type II TAL injury [11] were found in 2 and 7 patients, respectively.

**Table 1:** Clinical data of 13 patients

Case	Injury cause	Fracture characteristics	LMD (pre)	LMD (post)	VAS (pre)	VAS (post)	Axial ROM after surgery (°)	Bone union confirmed (month)	Follow-up (month)	Complication
1	MVA	Single AAF and single PAF	7.0	1.6	8	0	29.9	6	15	No
2	Falling	Single AAF and single PAF	4.7	0.0	7	0	43.2	6	12	No
3	MVA	Single AAF and single PAF	5.5	0.8	6	0	35.7	3	12	No
4	MVA	Double AAFs and single PAF	9.3	2.1	8	1	55.9	3	18	No
5	Falling	Single AAF and single PAF	7.2	1.4	7	2	60.3	9	20	No
6	Falling	Double AAFs and double PAFs	6.1	1.0	7	0	52.9	3	15	No
7	MVA	Single AAF and single PAF	3.5	0.0	6	0	39.4	6	24	No
8	MVA	Single AAF and single PAF	6.9	0.5	6	0	64.2	6	24	No
9	MVA	Double AAFs and single PAF	8.3	1.8	7	1	44.8	3	22	No
10	Falling	Single AAF and double PAFs	7.5	1.0	7	0	50.2	9	15	No
11	MVA	Single AAF and double PAFs	5.9	0.6	5	0	43.8	3	21	No
12	Falling	Single AAF and single PAF	5.5	1.2	6	0	62.3	6	16	No
13	MVA	Single AAF and single PAF	6.7	0.8	6	1	58.7	3	12	No
M ± SD			6.5 ± 1.5	1.0 ± 0.6	6.6 ± 0.9	0.4 ± 0.7	49.3 ± 10.8	5.1 ± 2.3	17.4 ± 4.4	
t			19.090		24.239					
P			0.000^a^		0.000^a^					

*M* Male, *F* Female, *MVA* Motor verhicle accident, *AAF* Anterior arch fracture, *PAF* Posterior arch fracture, *LMD* Lateral mass displacement, *VAS* Visual analog scale, *ROM* Range of motion

^a^ Paired-sample t-test

### Surgical procedure

Preoperative preparation: All patients were instructed to gargle six times per day with 0.02% chlorhexidine acetate prior to surgery. A professional dental cleaning was also performed prior to surgery. A nasogastric feeding tube was placed and prophylactic broad-spectrum antibiotics were applied conventionally 30 min before the operation.

**Fig. 1:**
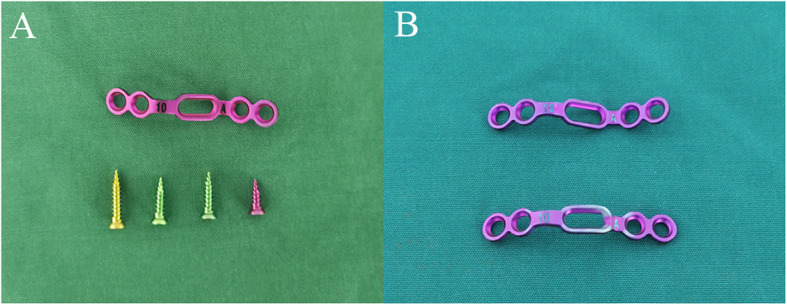
Posterior cervical laminoplasty screw-plate system. **a** Laminoplasty plate and tapping screws in different lengths. **b** Laminoplasty plates before and after shaping

**Fig. 2:**
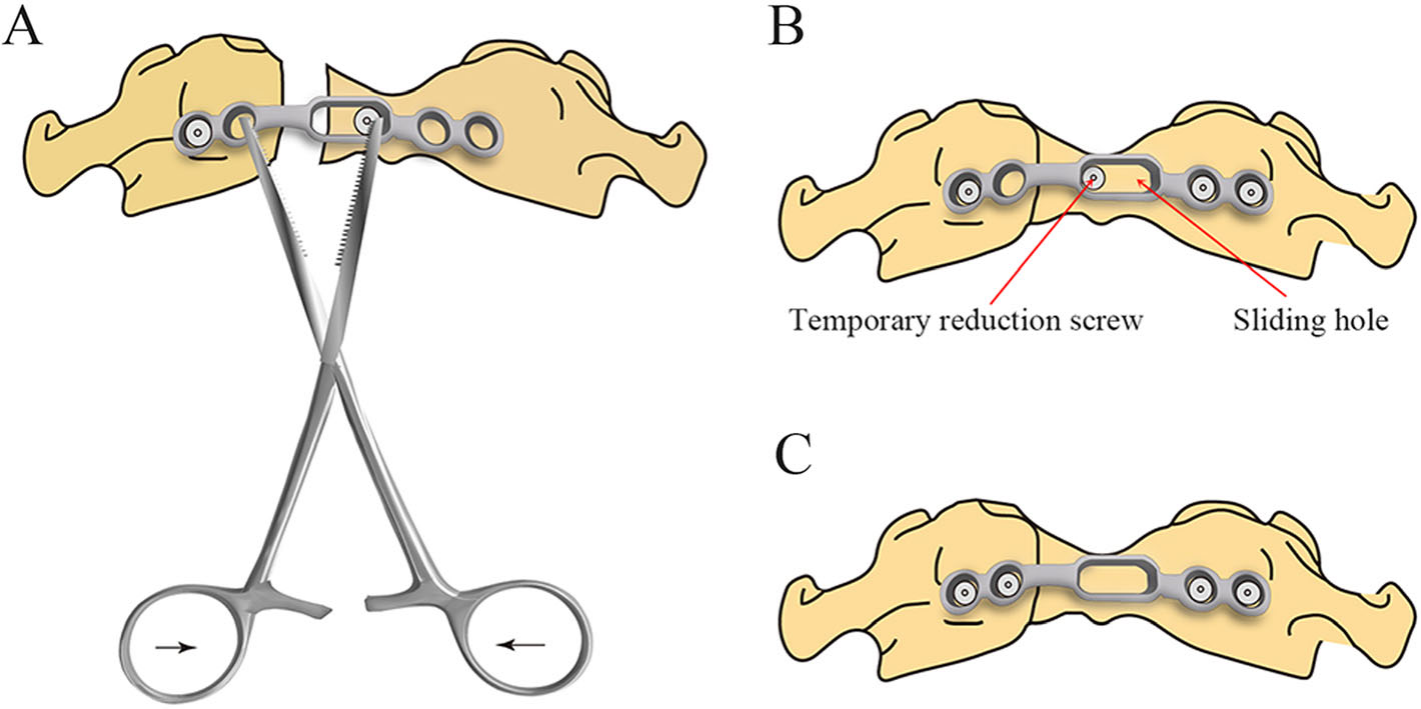
Reduction schematics. **a** The forceps handles were closed to impart a compression force to reset the fracture after placement of one side of plate and temporary reduction screw. **b** Reduction of fracture was achieved, and another side of plate was fixed using two screws. **c** The last screw was placed, and the temporary reduction screw was removed

Surgical technique: Under general anesthesia with nasotracheal intubation, the patient was positioned supine, and the neck was situated to be slightly hyperextended. After routine oral cleaning and disinfection, oral cavity was opened by a Codman retractor. The anatomic land marks in all transoral operations can be confirmed under the guidance of intraoperative fluoroscopy. Then, a longitudinal incision of 3–4 cm was made in the median posterior pharyngeal wall to incise the mucosa and split longitudinal muscles. After the anterior arch of C1 and the anterior aspect of lateral mass and fracture of the atlas were exposed, an appropriately sized plate (Posterior cervical laminoplasty screw-plate system, Fule, China; Fig. 1) was shaped to suit for anterior arch. If the anterior arch had single fracture, a side of plate was fixed on the lateral mass near the fracture gap using one screw firstly. After a temporary reduction screw was inserted into the anterior arch through the sliding hole of the plate, a Cook hemostatic forceps was installed between a hole and temporary reduction screw. Then, the forceps handles were closed to impart a compression force to restore the fracture (Fig. 2a). After the reduction of fracture was confirmed under direct vision, another side of plate was fixed using two screws (Fig. 2b). Then, the last hole was implanted with a screw, and the temporary reduction screw was removed (Fig. 2c). If the anterior arch had double fractures, the reduction of fractures was performed by inward extrusion on the lateral masses using a Crutchfield clamp [8], and then, an appropriately sized plate was placed in front of the atlas to fix the fractures directly. After the placement of the plate and screws was verified by C-arm fluoroscopy, the incision was closed in the muscular and mucosal layers.

### Postoperative management and follow-up

After surgery, the tracheal cannula was removed after 24–48 h, and the nasogastric feeding tube was removed after 7 days. Ultrasonic nebulization and 0.02% chlorhexidine acetate gargling were performed 3–5 times per day for 7 days. Cervical radiographs and CT scans were obtained 5 days postoperatively to evaluate fracture reduction and the position of fixation. The total lateral mass displacement (LMD) was also measured on the coronal CT images. Postoperative external immobilization with a rigid cervical collar was used for 4–6 weeks. Patients were followed up at 3, 6, 9 and 12 months and then once per year or whenever needed with assessment of neck pain on a visual analog scale (VAS), and with neurological status. Cervical radiographs and CT scans were performed at each follow-up. Bone union of fractures was evaluated by CT scan and confirmed by complete fusion of anterior arch fractures.

### Statistical analysis

All data were subject to normal distribution based on K-S test, which were expressed as the mean and standard deviation. The data were analyzed statistically with the paired-samples t-test, using SPSS 19.0 software (IBM, Armonk, NY, USA). The level of significance was set at *p* < 0.05.

## Results

**Fig.3:**
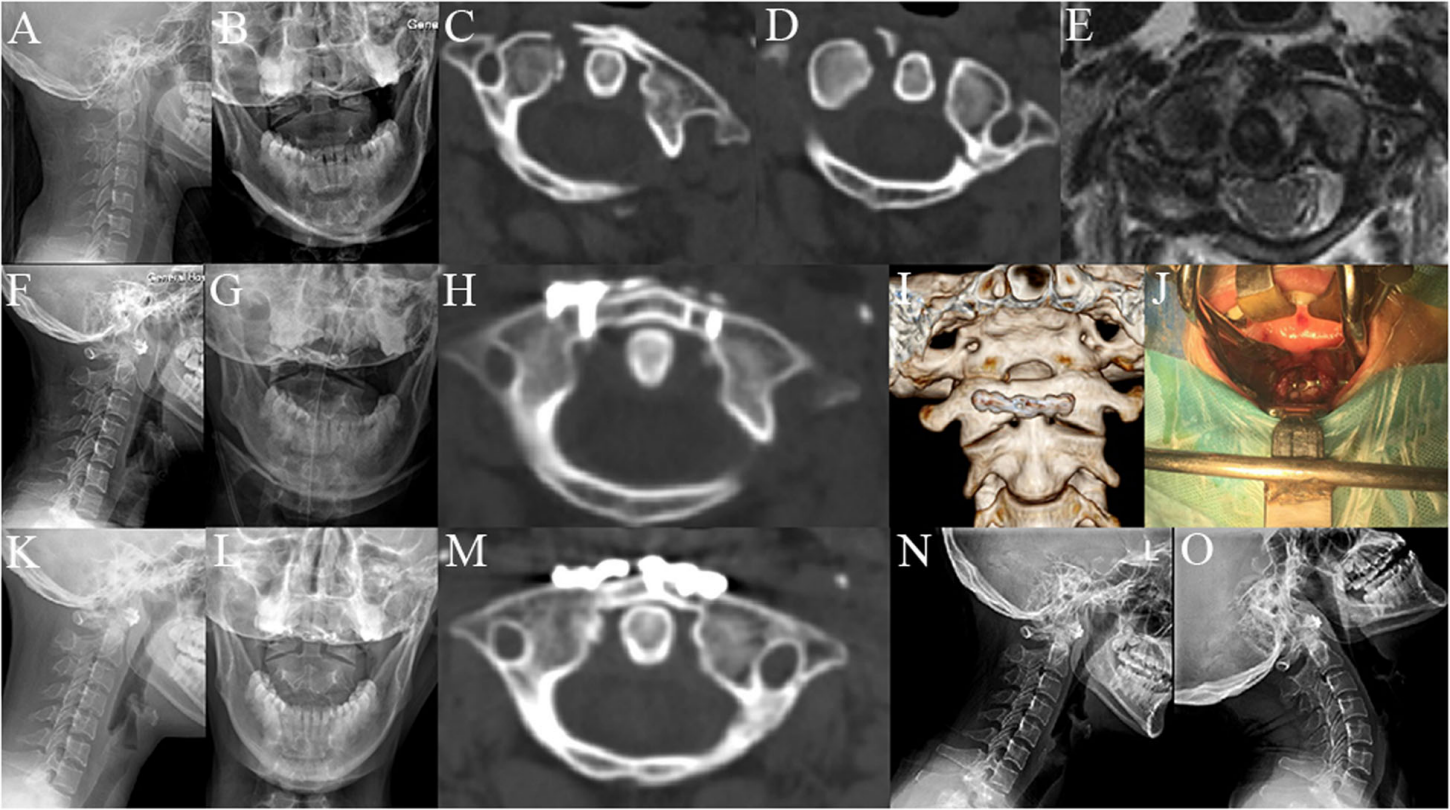
A 32-year-old female with atlas fracture treated by transoral anterior C1-ring osteosynthesis using a laminoplasty plate. Preoperative cervical lateral (**a**) and open-mouth X-rays (**b**), axial CT (**c**, **d**) and MRI images (**e**) showed combined fracture of the anterior and posterior arches with type I TAL injury. Postoperative cervical lateral (**f**) and open-mouth X-rays (**g**) identified the relatively good C1–C2 alignment. Axial CT (**h**) and the reconstructed images (**i**) after surgery showed reduction of the anterior arch fracture and optimal plate location. Transoral intraoperative view (**j**) showed good position of anterior laminoplasty plate fixation. Cervical lateral (**k**) and open-mouth X-rays (**l**) at 6 months after surgery showed stable fixation. An axial CT image (**m**) at 6 months after surgery indicated solid bone union. The dynamic cervical X-rays (**n**, **o**) at final follow-up revealed no sign of instability of C1–C2 and no loosening of the fixation

**Fig.4:**
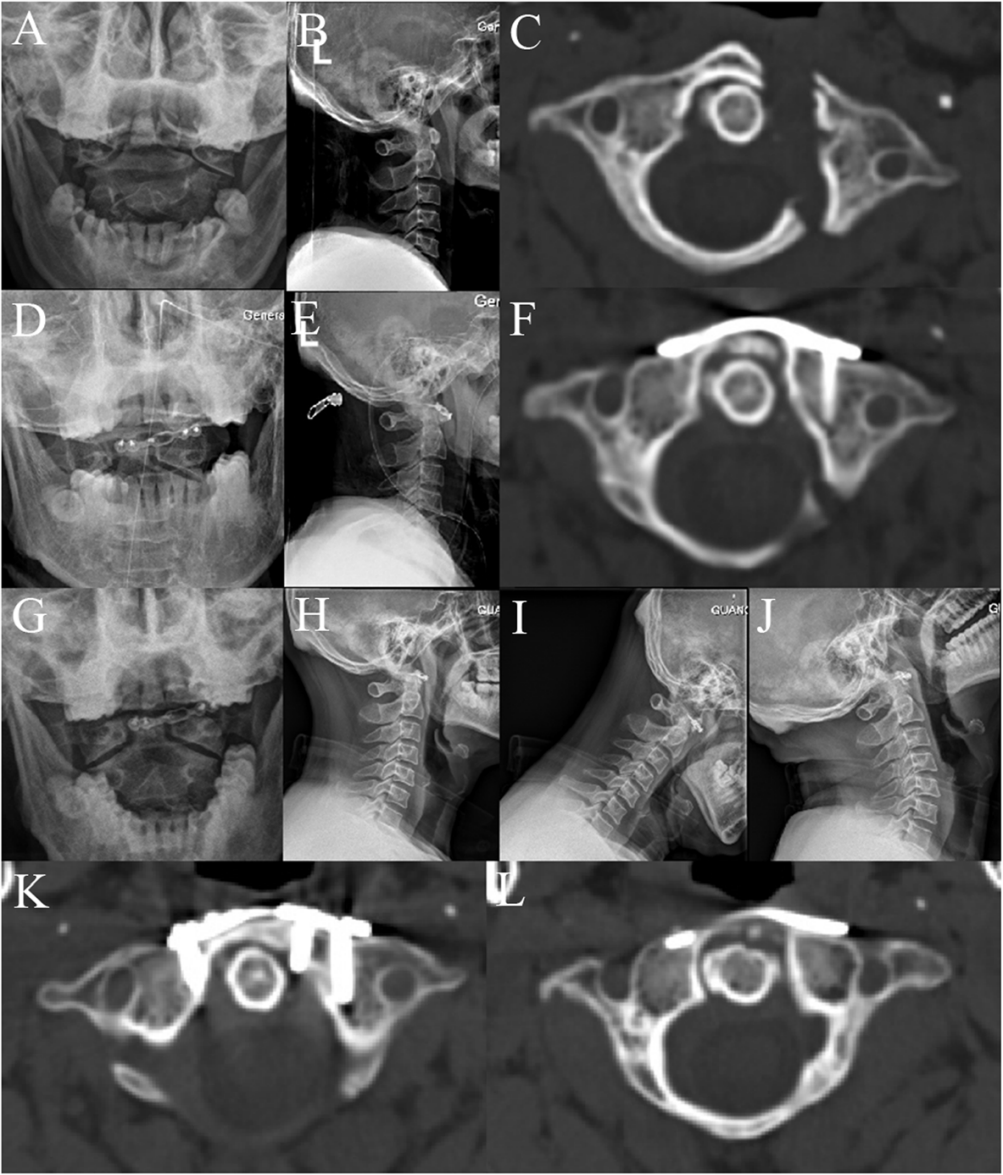
A 41-year-old female with atlas fracture treated by transoral anterior C1-ring osteosynthesis using a laminoplasty plate. Preoperative open-mouth (**a**) and cervical lateral X-rays (**b**), and axial CT (**c**) showed combined fracture of the anterior and posterior arches. Postoperative open-mouth (**d**) and cervical lateral X-rays (**e**) revealed the relatively good C1–C2 alignment. Axial CT (**f**) after surgery identified good reduction of the anterior and posterior arch fractures, and optimal placement of plate. Open-mouth (**g**), cervical lateral (**h**) and dynamic X-rays (**i**, **j**) at 9 months after surgery showed reliable fixation without instability of C1–C2. Axial CT images (**k**, **l**) at 9 months after surgery indicated satisfactory bone union of anterior and posterior arch fractures

The surgeries were performed successfully in all 13 cases. No damage to the spinal nerves, blood vessels, or dura mater occurred during the operations. The mean time of the procedure was 86.9 ± 16.7 min (ranging from 60 to 120 min), with an average intraoperative blood loss of 52.3 ± 16.9 mL (ranging from 30 to 80 mL). The average follow-up was 17.4 ± 4.4 months (ranging from 12 to 24 months). Postoperative CT scan showed well-placement of plates and screws, and satisfactory reduction of fractures of the anterior and posterior atlantal arches in all cases. The postoperative LMD (1.0 ± 0.6 mm, ranging from 0.0 to 2.1 mm) was significantly reduced compared with preoperative LMD (6.5 ± 1.5 mm, ranging from 3.5 to 9.3 mm; *p* < 0.01). One patient suffered screw loosening 1 week after operation and underwent replacement procedure subsequently. No internal fixation loosening or breakage was revealed following CT scans and plain radiographs during the follow-up. No complications of infection were noted. All patients achieved successful bone union of fractures after 3–9 months (Figs. 3 and 4). The axial range of motion was 49.3° ± 10.8° (ranging from 29.9° to 64.2°) at the final follow-up. All patients had a well-preserved range of motion of the upper cervical spine without signs of instability. Preoperative VAS scores (6.6 ± 0.9; range 5–8) had a markedly decrease (0.4 ± 0.7; range 0–2; *p* < 0.01) at the final follow-up (Table 1).

## Discussion

The atlas, the ring-shaped first cervical vertebra, does not have a vertebral body or spinous process. It is formed by the anterior and posterior arches and the two lateral masses. The weakest points of the atlas are the regions of the anterior and posterior arches that connect with the lateral masses, which are most likely to be injured with two or more fractures in the ring structure [12]. The stability of atlas fractures has historically been based primarily on the integrity of the TAL [2]. But, it is now believed that combined fractures of the anterior and posterior atlantal arches are the unstable fractures, whether or not they are associated with rupture of the TAL [3, 4, 8].

Symptoms of neurological dysfunction are rare in patients with atlas fracture because fractures of the atlas ring increase the space that is available for the dural sac, thereby inhibiting compression [13]. So that, stabilization of fractures is the most important factor for the treatment of atlas fractures. Although there is an agreement regarding treatment of stable atlas fractures, the optimal management of unstable atlas fractures remains controversial. Previously, nonsurgical treatments with skull traction, followed by external halo-vest immobilization, has been commonly suggested [14]. Most patients are can be treated by external immobilization with satisfactory outcomes. However, a follow-up study of 22 patients with atlas burst fractures by Dvorak et al. [15] showed that patients that underwent conservative treatments failed to regain functional preoperative levels, and hinted that nonsurgical treatments were not optimal management options. The strongest Halo-vest has only 75% restriction on cervical flexion and extension activity, so that, preservative treatment with a Halo-vest has a high risk of nonunion [5]. Immobilization of the cervical spine for several months may result in significant discomfort and other complications especially in elderly patients [16]. Moreover, mechanical instability and incongruence of the atlanto-occipital and the atlanto-axial joints may lead to arthrosis, persistent neck pain, and even neurologic injury.

Posterior C1–C2 or C0–C2 fixation and fusion techniques are widely used in unstable atlas fractures, including C1–C2 transarticular screw fixation, C1–C2 screw-rod fixation, and C0–C2 plate-screw-rod fixation [17, 18]. These fixation techniques promote biomechanical stability and guarantee a high bone fusion rate [19–22]. However, these treatments sacrifice the normal motion of the C1–C2 joints and possible increase the incidence of subaxial cervical spine degeneration [2].

In 2004, Ruf et al. [7] firstly reported a motion-preserving technique, C1-ring osteosynthesis, using a lateral mass screw-rod construct by transoral approach for unstable atlas fractures, with favorable clinical outcomes. Dickman hypothesizes that permanent anterior instability of C1–C2 results from TAL rupture [23]. However, biomechanical studies showed that within the physiological loading range, the longitudinal ligaments had sufficient capacity to maintain the stability of the atlantoaxial joint even with concomitant TAL injuries in atlas fractures [24, 25]. C1-ring osteosynthesis may therefore be a valid alternative to C1–C2 fusion in the treatment of unstable atlas fractures even with TAL rupture [7, 8, 26, 27]. But, a lateral mass screw-rod construct used by Ruf et al. [7] was not suitable for anterior anatomy of the atlas. Ma et al. [8] and Hu et al. [9] used a reconstruction plate in transoral anterior C1-ring osteosynthesis for unstable atlas fractures, but this plate did not have reduction mechanism. Thus, we performed transoral anterior C1-ring osteosynthesis for unstable atlas fractures using a laminoplasty plate which has a reduction function, that can restore fractures and preserve normal C1–C2 motion.

In our study, 13 patients with an unstable atlas fracture were treated with a laminoplasty plate C1-ring osteosynthesis by transoral anterior approach and were followed-up to assess the preliminary efficacy of this technique. This technique can not only reduce the fracture of anterior atlantal arches, but also reduce the dislocation of lateral mass, and at the same time, the fracture of posterior atlantal arches reduced automatically. All cases achieved satisfactory reduction of fractures, well-preserved range of motion and satisfactory bone union without signs of instability or complications. Wound infection can be a serious problem for the transoral anterior approach. But with proper preoperative preparation and postoperative care, the infection can be reduced and even prevented [8, 28]. In this study, no signs and symptoms of infection were found after surgery and during the follow-up.

There are several limitations in the current study. First, the small sample size is the primary limitation. The safety and efficacy of this technique need to be evaluated with more cases. In addition, the present study is retrospective in nature; future prospective studies may better control for follow-up timing intervals and may have the potential to include more standardized outcome measures.

## Conclusions

Transoral anterior C1-ring osteosynthesis using a laminoplasty plate is a effective surgical option to manage unstable atlas fractures. This technique can provide satisfactory reduction, reliable stabilization and bone union of fracture, and preserve important C1–C2 motion.

## Data Availability

The data used and analyzed during the current study are available in anonymized form from the corresponding author on reasonable request.

## Abbreviations

TAL: Transverse atlantal ligament
CT: Computed tomography
MRI: Magnetic resonance imaging
LMD: Lateral mass displacement
VAS: Visual analog scale
MVA: Motor verhicle accident
AAF: Anterior arch fracture
PAF: Posterior arch fracture
ROM: Range of motion
